# 1-Acetyl-*c*-3,*t*-3-dimethyl-*r*-2,*c*-6-diphenyl­piperidin-4-one

**DOI:** 10.1107/S1600536809028049

**Published:** 2009-07-25

**Authors:** S. Aravindhan, S. Ponnuswamy, M. Jamesh, P. Ramesh, M. N. Ponnuswamy

**Affiliations:** a Department of Physics, Presidency College (Autonomous), Chennai 600 005, India; b Department of Chemistry, Government Arts College (Autonomous), Coimbatore 641 018, India; c Centre of Advanced Study in Crystallography and Biophysics, University of Madras, Guindy Campus, Chennai 600 025, India

## Abstract

In the title compound, C_21_H_23_NO_2_, the piperidine ring adopts a distorted boat conformation. The two phenyl rings form dihedral angles of 64.6 (1) and 87.8 (1)° with the best plane through the piperidine ring. The crystal packing is governed by inter­molecular C—H⋯O inter­actions.

## Related literature

For the biological activity of piperidine derivatives, see: Ponnuswamy *et al.* (2002 ▶). For hydrogen-bond motifs, see: Bernstein *et al.* (1995 ▶). For puckering and asymmetry parameters, see: Cremer & Pople (1975 ▶); Nardelli (1983 ▶).
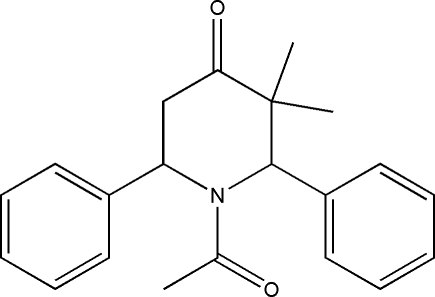


## Experimental

### 

#### Crystal data

C_21_H_23_NO_2_
*M* *_r_* = 321.40
Monoclinic, 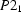

*a* = 7.5622 (4) Å
*b* = 10.6369 (5) Å
*c* = 11.1497 (7) Å
β = 100.373 (3)°
*V* = 882.21 (8) Å^3^
*Z* = 2
Mo *K*α radiation
μ = 0.08 mm^−1^
*T* = 293 K
0.30 × 0.25 × 0.20 mm

#### Data collection

Bruker Kappa APEXII area-detector diffractometer
Absorption correction: multi-scan (*SADABS*; Sheldrick, 2001 ▶) *T*_min_ = 0.977, *T*_max_ = 0.985
12819 measured reflections
3457 independent reflections
2400 reflections with *I* > 2σ(*I*)
*R*_int_ = 0.042

#### Refinement

*R*[*F* ^2^ > 2σ(*F* ^2^)] = 0.046
*wR*(*F* ^2^) = 0.145
*S* = 1.02
3457 reflections
220 parameters
1 restraint
H-atom parameters constrained
Δρ_max_ = 0.23 e Å^−3^
Δρ_min_ = −0.17 e Å^−3^

### 

Data collection: *APEX2* (Bruker, 2004 ▶); cell refinement: *SAINT* (Bruker, 2004 ▶); data reduction: *SAINT*; program(s) used to solve structure: *SHELXS97* (Sheldrick, 2008 ▶); program(s) used to refine structure: *SHELXL97* (Sheldrick, 2008 ▶); molecular graphics: *ORTEP-3* (Farrugia, 1997 ▶); software used to prepare material for publication: *SHELXL97* and *PLATON* (Spek, 2009 ▶).

## Supplementary Material

Crystal structure: contains datablocks global, I. DOI: 10.1107/S1600536809028049/bt2989sup1.cif

Structure factors: contains datablocks I. DOI: 10.1107/S1600536809028049/bt2989Isup2.hkl

Additional supplementary materials:  crystallographic information; 3D view; checkCIF report

## Figures and Tables

**Table 1:** Hydrogen-bond geometry (Å, °)

*D*—H⋯*A*	*D*—H	H⋯*A*	*D*⋯*A*	*D*—H⋯*A*
C8—H8*A*⋯O2^i^	0.96	2.53	3.442 (3)	159
C14—H14⋯O1^ii^	0.93	2.39	3.124 (3)	135

Symmetry codes: (i) 
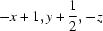
; (ii) 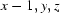.

**Fig. 1.**
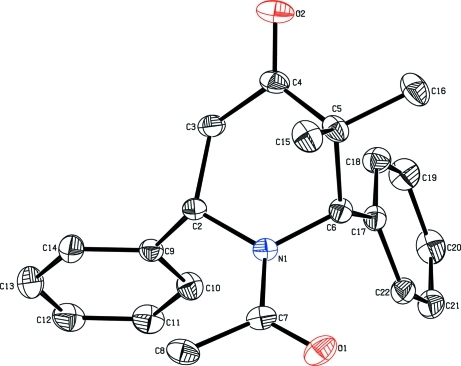
Perspective view of the molecule with displacement ellipsoids at the 50% probability level. The H atoms are omitted for clarity.

**Fig. 2.**
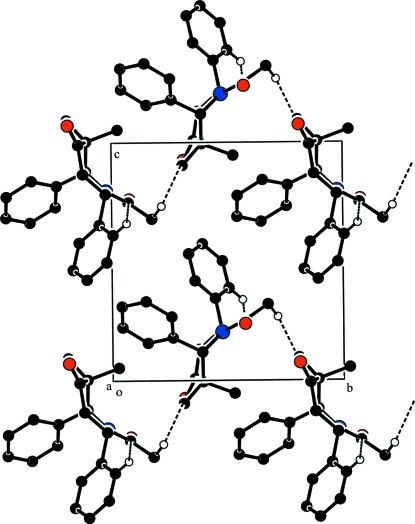
The crystal packing viewed down a axis. H atoms not involved in hydrogen bonding have been omitted for clarity.

## supplementary crystallographic information

### Comment

The design and synthesis of conformationally anchored molecules are important due its potency and selectivity for designing drugs. The piperidin-4-ones are one such class of compounds to be investigated to understand the stereodynamics and other structural features (Ponnuswamy *et al.*, 2002). In view of these importance and to ascertain the molecular conformation, crystallographic study of the title compound has been carried out.

The *ORTEP* diagram of the title compound is shown in Fig.1. The piperidine ring adopts a distorted boat conformation. The puckering parameters (Cremer & Pople, 1975) and the asymmetry parameters (Nardelli, 1983) for this ring are q_2_ = 0.592 (2) Å, q_3_ = 0.116 (2) Å, π = 284.8 (2)° and Δs(C3) =Δs(C6)= 15.2 (2)°. The sum of the angles at N1 (359.03°) is in accrdance with *sp*^2^ hybridization. The two phenyl rings are twisted away from the best plane of the pyridine ring by 64.6 (1)° and 87.8 (1)°, respectively.

The crystal packing is controlled by C—H···O types of intra and intermolecular interactions in addition to van der Waals forces. Atom C8 at (*x*, *y*, *z*) donates a proton to O2 (1 - *x*,1/2 + *y*,-*z*), which forms a C(8) (Bernstein, *et al.*, 1995) zigzag chain running along *b* axis shown in Fig. 2.

### Experimental

A mixture of c-3,t-3-dimethyl-r-2,c-6-diphenylpiperidin-4-one (1.4 g, 5 mmol), acetyl chloride (0.7 ml, 10 mmol) and triethylamine (2 ml, 14.4 mmol) in anhydrous benzene (50 ml) was stirred at room temparature for 7 h. The precipitated ammonium salt was filtered off and the filtrate was washed with water (4x10ml). The resulting pasty mass was purified and crystallized from benzene and pet-ether (60–80°C) in the ratio of 95: 5.

### Refinement

In the absence of anomalous scatterers Friedel pairs were merged and the absolute configuration was arbitrarily set. All H atoms were positioned geometrically (C—H=0.93–0.98 Å) and allowed to ride on their parent atoms, with 1.5*U*_eq_(C) for methyl H and 1.2 *U*_eq_(C) for other H atoms.

### Crystal data

**Table:**

C_21_H_23_NO_2_	*F*(000) = 344
*M**_r_* = 321.40	*D*_x_ = 1.210 Mg m^−^^3^
Monoclinic, *P*2_1_	Mo *K*α radiation, λ = 0.71073 Å
Hall symbol: P 2yb	Cell parameters from 3546 reflections
*a* = 7.5622 (4) Å	θ = 1.9–34.8°
*b* = 10.6369 (5) Å	µ = 0.08 mm^−^^1^
*c* = 11.1497 (7) Å	*T* = 293 K
β = 100.373 (3)°	Block, colorless
*V* = 882.21 (8) Å^3^	0.30 × 0.25 × 0.20 mm
*Z* = 2	

### Data collection

**Table:**

Bruker Kappa APEXII area-detector diffractometer	3457 independent reflections
Radiation source: fine-focus sealed tube	2400 reflections with *I* > 2σ(*I*)
graphite	*R*_int_ = 0.042
ω and φ scans	θ_max_ = 34.8°, θ_min_ = 1.9°
Absorption correction: multi-scan (*SADABS*; Sheldrick, 2001)	*h* = −11→11
*T*_min_ = 0.977, *T*_max_ = 0.985	*k* = −17→11
12819 measured reflections	*l* = −16→16

### Refinement

**Table:**

Refinement on *F*^2^	Primary atom site location: structure-invariant direct methods
Least-squares matrix: full	Secondary atom site location: difference Fourier map
*R*[*F*^2^ > 2σ(*F*^2^)] = 0.046	Hydrogen site location: inferred from neighbouring sites
*wR*(*F*^2^) = 0.145	H-atom parameters constrained
*S* = 1.02	*w* = 1/[σ^2^(*F*_o_^2^) + (0.0853*P*)^2^] where *P* = (*F*_o_^2^ + 2*F*_c_^2^)/3
3457 reflections	(Δ/σ)_max_ = 0.012
220 parameters	Δρ_max_ = 0.23 e Å^−^^3^
1 restraint	Δρ_min_ = −0.17 e Å^−^^3^

### Special details

**Table:**

Geometry. All e.s.d.'s (except the e.s.d. in the dihedral angle between two l.s. planes) are estimated using the full covariance matrix. The cell e.s.d.'s are taken into account individually in the estimation of e.s.d.'s in distances, angles and torsion angles; correlations between e.s.d.'s in cell parameters are only used when they are defined by crystal symmetry. An approximate (isotropic) treatment of cell e.s.d.'s is used for estimating e.s.d.'s involving l.s. planes.
Refinement. Refinement of *F*^2^ against ALL reflections. The weighted *R*-factor *wR* and goodness of fit *S* are based on *F*^2^, conventional *R*-factors *R* are based on *F*, with *F* set to zero for negative *F*^2^. The threshold expression of *F*^2^ > σ(*F*^2^) is used only for calculating *R*-factors(gt) *etc*. and is not relevant to the choice of reflections for refinement. *R*-factors based on *F*^2^ are statistically about twice as large as those based on *F*, and *R*- factors based on ALL data will be even larger.

### Fractional atomic coordinates and isotropic or equivalent isotropic displacement parameters (Å^2^)

**Table:**

	*x*	*y*	*z*	*U*_iso_*/*U*_eq_	
O1	1.0626 (2)	0.57532 (19)	0.23859 (18)	0.0680 (5)	
O2	0.4399 (3)	0.3113 (2)	−0.07440 (15)	0.0763 (5)	
N1	0.7953 (2)	0.47573 (14)	0.20329 (14)	0.0393 (3)	
C2	0.6053 (2)	0.47297 (17)	0.21592 (15)	0.0392 (3)	
H2	0.5538	0.5559	0.1928	0.047*	
C3	0.4980 (3)	0.3754 (2)	0.13125 (18)	0.0493 (4)	
H3A	0.5087	0.2952	0.1735	0.059*	
H3B	0.3722	0.3993	0.1187	0.059*	
C4	0.5484 (3)	0.3556 (2)	0.00799 (18)	0.0507 (5)	
C5	0.7355 (3)	0.3903 (2)	−0.00532 (17)	0.0492 (4)	
C6	0.8617 (3)	0.38858 (19)	0.11952 (16)	0.0414 (4)	
H6	0.9759	0.4240	0.1055	0.050*	
C7	0.9060 (3)	0.57188 (19)	0.25158 (18)	0.0464 (4)	
C8	0.8306 (4)	0.6735 (2)	0.3207 (2)	0.0600 (5)	
H8A	0.7347	0.7155	0.2675	0.090*	
H8B	0.9234	0.7330	0.3509	0.090*	
H8C	0.7852	0.6371	0.3879	0.090*	
C9	0.5778 (2)	0.44554 (17)	0.34515 (16)	0.0406 (4)	
C10	0.6819 (3)	0.3583 (2)	0.41885 (18)	0.0508 (4)	
H10	0.7759	0.3178	0.3912	0.061*	
C11	0.6462 (4)	0.3315 (2)	0.5329 (2)	0.0606 (6)	
H11	0.7158	0.2726	0.5819	0.073*	
C12	0.5085 (4)	0.3913 (3)	0.5744 (2)	0.0670 (7)	
H12	0.4851	0.3732	0.6517	0.080*	
C13	0.4058 (4)	0.4773 (3)	0.5026 (3)	0.0726 (7)	
H13	0.3123	0.5175	0.5310	0.087*	
C14	0.4399 (3)	0.5051 (2)	0.3878 (2)	0.0571 (5)	
H14	0.3696	0.5641	0.3393	0.069*	
C15	0.7232 (4)	0.5268 (2)	−0.0542 (2)	0.0616 (6)	
H15A	0.6699	0.5795	−0.0005	0.092*	
H15B	0.6505	0.5286	−0.1341	0.092*	
H15C	0.8416	0.5571	−0.0581	0.092*	
C16	0.8096 (4)	0.3069 (3)	−0.0963 (2)	0.0699 (7)	
H16A	0.7373	0.3168	−0.1758	0.105*	
H16B	0.8067	0.2206	−0.0714	0.105*	
H16C	0.9313	0.3308	−0.0988	0.105*	
C17	0.9083 (2)	0.26144 (18)	0.17969 (16)	0.0417 (4)	
C18	0.8065 (3)	0.1533 (2)	0.1580 (2)	0.0525 (5)	
H18	0.7036	0.1538	0.0981	0.063*	
C19	0.8543 (4)	0.0441 (2)	0.2235 (2)	0.0634 (6)	
H19	0.7835	−0.0276	0.2077	0.076*	
C20	1.0062 (4)	0.0418 (2)	0.3117 (2)	0.0654 (6)	
H20	1.0375	−0.0309	0.3570	0.079*	
C21	1.1104 (3)	0.1459 (3)	0.3327 (2)	0.0600 (6)	
H21	1.2149	0.1434	0.3912	0.072*	
C22	1.0634 (3)	0.2562 (2)	0.26817 (18)	0.0500 (4)	
H22	1.1360	0.3270	0.2842	0.060*	

### Atomic displacement parameters (Å^2^)

**Table:**

	*U*^11^	*U*^22^	*U*^33^	*U*^12^	*U*^13^	*U*^23^
O1	0.0472 (8)	0.0620 (10)	0.0923 (12)	−0.0134 (8)	0.0056 (8)	0.0097 (9)
O2	0.0845 (12)	0.0894 (13)	0.0451 (9)	−0.0115 (11)	−0.0149 (8)	−0.0095 (9)
N1	0.0402 (7)	0.0397 (7)	0.0373 (7)	−0.0037 (6)	0.0051 (6)	0.0018 (6)
C2	0.0390 (8)	0.0421 (8)	0.0349 (8)	−0.0003 (7)	0.0023 (6)	0.0005 (6)
C3	0.0429 (9)	0.0589 (12)	0.0433 (10)	−0.0061 (8)	−0.0001 (7)	−0.0048 (8)
C4	0.0613 (12)	0.0512 (10)	0.0343 (9)	0.0020 (9)	−0.0058 (8)	0.0008 (7)
C5	0.0658 (12)	0.0487 (10)	0.0327 (8)	0.0094 (9)	0.0081 (8)	0.0039 (7)
C6	0.0429 (8)	0.0453 (9)	0.0368 (8)	0.0025 (7)	0.0093 (7)	0.0054 (7)
C7	0.0499 (10)	0.0398 (9)	0.0456 (9)	−0.0071 (8)	−0.0018 (8)	0.0104 (7)
C8	0.0766 (14)	0.0449 (11)	0.0548 (12)	−0.0111 (10)	0.0022 (10)	−0.0039 (9)
C9	0.0403 (8)	0.0428 (9)	0.0387 (8)	−0.0052 (7)	0.0069 (7)	−0.0029 (7)
C10	0.0585 (11)	0.0520 (10)	0.0434 (10)	0.0020 (9)	0.0134 (8)	0.0051 (8)
C11	0.0724 (14)	0.0623 (13)	0.0475 (11)	−0.0077 (11)	0.0120 (10)	0.0099 (10)
C12	0.0712 (14)	0.0869 (17)	0.0465 (11)	−0.0237 (13)	0.0207 (11)	−0.0022 (11)
C13	0.0619 (13)	0.0986 (19)	0.0638 (15)	−0.0010 (15)	0.0287 (12)	−0.0103 (14)
C14	0.0493 (10)	0.0683 (14)	0.0547 (12)	0.0055 (10)	0.0118 (9)	−0.0031 (10)
C15	0.0848 (16)	0.0563 (12)	0.0443 (11)	0.0116 (11)	0.0129 (11)	0.0159 (9)
C16	0.0966 (19)	0.0713 (14)	0.0444 (12)	0.0180 (14)	0.0198 (12)	−0.0011 (11)
C17	0.0436 (9)	0.0458 (9)	0.0364 (8)	0.0071 (8)	0.0093 (7)	0.0044 (7)
C18	0.0543 (11)	0.0496 (10)	0.0504 (11)	0.0035 (9)	0.0011 (9)	0.0042 (8)
C19	0.0725 (14)	0.0477 (12)	0.0696 (15)	0.0031 (11)	0.0117 (12)	0.0106 (10)
C20	0.0843 (16)	0.0561 (13)	0.0573 (13)	0.0203 (12)	0.0163 (12)	0.0162 (10)
C21	0.0579 (12)	0.0752 (15)	0.0446 (11)	0.0216 (11)	0.0027 (9)	0.0064 (10)
C22	0.0463 (10)	0.0573 (11)	0.0460 (10)	0.0080 (9)	0.0068 (8)	0.0006 (9)

### Geometric parameters (Å, °)

**Table:**

O1—C7	1.219 (3)	C11—C12	1.370 (4)
O2—C4	1.211 (3)	C11—H11	0.9300
N1—C7	1.369 (2)	C12—C13	1.362 (4)
N1—C6	1.467 (2)	C12—H12	0.9300
N1—C2	1.469 (2)	C13—C14	1.383 (4)
C2—C9	1.521 (2)	C13—H13	0.9300
C2—C3	1.534 (3)	C14—H14	0.9300
C2—H2	0.9800	C15—H15A	0.9600
C3—C4	1.506 (3)	C15—H15B	0.9600
C3—H3A	0.9700	C15—H15C	0.9600
C3—H3B	0.9700	C16—H16A	0.9600
C4—C5	1.495 (3)	C16—H16B	0.9600
C5—C16	1.528 (3)	C16—H16C	0.9600
C5—C6	1.540 (3)	C17—C18	1.381 (3)
C5—C15	1.548 (3)	C17—C22	1.391 (3)
C6—C17	1.523 (3)	C18—C19	1.385 (3)
C6—H6	0.9800	C18—H18	0.9300
C7—C8	1.499 (3)	C19—C20	1.371 (4)
C8—H8A	0.9600	C19—H19	0.9300
C8—H8B	0.9600	C20—C21	1.355 (4)
C8—H8C	0.9600	C20—H20	0.9300
C9—C14	1.376 (3)	C21—C22	1.389 (3)
C9—C10	1.387 (3)	C21—H21	0.9300
C10—C11	1.376 (3)	C22—H22	0.9300
C10—H10	0.9300		
			
C7—N1—C6	117.82 (15)	C9—C10—H10	120.0
C7—N1—C2	121.12 (15)	C12—C11—C10	120.3 (2)
C6—N1—C2	120.09 (15)	C12—C11—H11	119.9
N1—C2—C9	113.43 (14)	C10—C11—H11	119.9
N1—C2—C3	111.90 (14)	C13—C12—C11	120.0 (2)
C9—C2—C3	107.80 (15)	C13—C12—H12	120.0
N1—C2—H2	107.8	C11—C12—H12	120.0
C9—C2—H2	107.8	C12—C13—C14	120.4 (2)
C3—C2—H2	107.8	C12—C13—H13	119.8
C4—C3—C2	117.60 (17)	C14—C13—H13	119.8
C4—C3—H3A	107.9	C9—C14—C13	120.0 (2)
C2—C3—H3A	107.9	C9—C14—H14	120.0
C4—C3—H3B	107.9	C13—C14—H14	120.0
C2—C3—H3B	107.9	C5—C15—H15A	109.5
H3A—C3—H3B	107.2	C5—C15—H15B	109.5
O2—C4—C5	123.0 (2)	H15A—C15—H15B	109.5
O2—C4—C3	119.9 (2)	C5—C15—H15C	109.5
C5—C4—C3	117.11 (17)	H15A—C15—H15C	109.5
C4—C5—C16	112.9 (2)	H15B—C15—H15C	109.5
C4—C5—C6	110.54 (15)	C5—C16—H16A	109.5
C16—C5—C6	110.58 (18)	C5—C16—H16B	109.5
C4—C5—C15	105.60 (19)	H16A—C16—H16B	109.5
C16—C5—C15	108.50 (18)	C5—C16—H16C	109.5
C6—C5—C15	108.51 (18)	H16A—C16—H16C	109.5
N1—C6—C17	111.05 (14)	H16B—C16—H16C	109.5
N1—C6—C5	109.90 (15)	C18—C17—C22	117.57 (18)
C17—C6—C5	117.75 (17)	C18—C17—C6	125.84 (17)
N1—C6—H6	105.7	C22—C17—C6	116.52 (18)
C17—C6—H6	105.7	C17—C18—C19	121.5 (2)
C5—C6—H6	105.7	C17—C18—H18	119.3
O1—C7—N1	120.9 (2)	C19—C18—H18	119.3
O1—C7—C8	120.5 (2)	C20—C19—C18	119.9 (2)
N1—C7—C8	118.58 (18)	C20—C19—H19	120.1
C7—C8—H8A	109.5	C18—C19—H19	120.1
C7—C8—H8B	109.5	C21—C20—C19	119.7 (2)
H8A—C8—H8B	109.5	C21—C20—H20	120.1
C7—C8—H8C	109.5	C19—C20—H20	120.1
H8A—C8—H8C	109.5	C20—C21—C22	121.0 (2)
H8B—C8—H8C	109.5	C20—C21—H21	119.5
C14—C9—C10	119.22 (17)	C22—C21—H21	119.5
C14—C9—C2	118.71 (18)	C21—C22—C17	120.4 (2)
C10—C9—C2	122.00 (16)	C21—C22—H22	119.8
C11—C10—C9	120.1 (2)	C17—C22—H22	119.8
C11—C10—H10	120.0		
			
C7—N1—C2—C9	−70.9 (2)	C6—N1—C7—C8	170.20 (17)
C6—N1—C2—C9	120.55 (17)	C2—N1—C7—C8	1.4 (3)
C7—N1—C2—C3	166.82 (16)	N1—C2—C9—C14	143.64 (19)
C6—N1—C2—C3	−1.7 (2)	C3—C2—C9—C14	−91.9 (2)
N1—C2—C3—C4	−35.4 (2)	N1—C2—C9—C10	−39.4 (2)
C9—C2—C3—C4	−160.78 (17)	C3—C2—C9—C10	85.1 (2)
C2—C3—C4—O2	−157.6 (2)	C14—C9—C10—C11	0.4 (3)
C2—C3—C4—C5	23.6 (3)	C2—C9—C10—C11	−176.5 (2)
O2—C4—C5—C16	−31.1 (3)	C9—C10—C11—C12	−0.4 (4)
C3—C4—C5—C16	147.7 (2)	C10—C11—C12—C13	0.3 (4)
O2—C4—C5—C6	−155.5 (2)	C11—C12—C13—C14	−0.2 (4)
C3—C4—C5—C6	23.3 (3)	C10—C9—C14—C13	−0.3 (3)
O2—C4—C5—C15	87.3 (3)	C2—C9—C14—C13	176.7 (2)
C3—C4—C5—C15	−93.9 (2)	C12—C13—C14—C9	0.2 (4)
C7—N1—C6—C17	107.34 (18)	N1—C6—C17—C18	104.0 (2)
C2—N1—C6—C17	−83.8 (2)	C5—C6—C17—C18	−23.9 (3)
C7—N1—C6—C5	−120.60 (18)	N1—C6—C17—C22	−73.0 (2)
C2—N1—C6—C5	48.3 (2)	C5—C6—C17—C22	159.13 (16)
C4—C5—C6—N1	−58.1 (2)	C22—C17—C18—C19	1.4 (3)
C16—C5—C6—N1	176.13 (18)	C6—C17—C18—C19	−175.6 (2)
C15—C5—C6—N1	57.2 (2)	C17—C18—C19—C20	−0.3 (4)
C4—C5—C6—C17	70.3 (2)	C18—C19—C20—C21	−1.2 (4)
C16—C5—C6—C17	−55.4 (2)	C19—C20—C21—C22	1.6 (4)
C15—C5—C6—C17	−174.30 (17)	C20—C21—C22—C17	−0.5 (3)
C6—N1—C7—O1	−9.6 (3)	C18—C17—C22—C21	−1.0 (3)
C2—N1—C7—O1	−178.35 (18)	C6—C17—C22—C21	176.26 (18)

### Hydrogen-bond geometry (Å, °)

**Table:**

*D*—H···*A*	*D*—H	H···*A*	*D*···*A*	*D*—H···*A*
C6—H6···O1	0.98	2.21	2.700 (3)	110
C8—H8A···O2^i^	0.96	2.53	3.442 (3)	159
C14—H14···O1^ii^	0.93	2.39	3.124 (3)	135

Symmetry codes:  (i) −*x*+1, *y*+1/2, −*z*; (ii) *x*−1, *y*, *z*.
